# 

**Published:** 1896-09

**Authors:** 


### CONTENTS.

ORIGINAL ARTICLES:
Extermination of the Cattle-tick. By COOPER CURTICE, D.V.M. . . . . 649
Navicular Disease (Navicular Arthritis). By H. L. DRAKE, D.V.S. . . . 655
Address of Retiring President Harbaugh of Virginia State Veterinary Medical Association . . . . . . . . . . . . . . . . 659
Experience with Barium Chloride. By JOS. PLASKETT, D.V.S. . . . , . 662
ABSTRACTS FROM FOREIGN JOURNALS:
Serum Therapeutics of Tetanus—Conference of the Slaughter-house Directors and Representatives of the Cities with Public Cattle-yards in Berlin—Internal Inflammation of the Eye of the Horse—Establishment of a Veterinary School in Upper Austria—Bohemian Veterinary Institution at Prague—Indemnification of Owners for Loss of Animals with Blackleg—Tyrol Petitions for a Veterinary School—Admission of Women to the Study of Veterinary Medicine—Cure of Cancer—Rye Bread with an Admixture of Malt as Food for Horses—Vomiting Among Cattle—Cataract in the Horse—Arecolin . . . . . . 667–678
REPORTS OF CASES:
A Case of Subnormal Temperature. By W. H. HARBAUGH, V.S. . . . . 679
Fluid Extract of Gelsemium in Tetanus. By W. G. HOLLINGSWORTH, D.V.S. . 680
EDITORIAL:
Rabies—The Campaign of Education—Don't Monkey with a Buzz-saw—After Buffalo, Where? . . . . . . . . . . . . . . 681–684
NECROLOGY—John W. Gadsden—George Leich . . . . . . . 685
AFTERMATH . . . . . . . . . . . . . . . . . 686
THE BUFFALO RESOLUTIONS . . . . . . . . . . . . 691
CONTROL WORK:
California—New Hampshire—Vermont—Connecticut—New York—Pennsylvania—Ohio — Indiana — Illinois — Michigan — Missouri — Oklahoma—Dominion of Canada . . . . . . . . . . . . . . . . 694–699
SOCIETY PROCEEDINGS:
U. S. V. M. A.—Maine Veterinary Medical Association—New Hampshire Veterinary Medical Association—Oneida County Veterinary Medical Association—Keystone Veterinary Medical Association—Pennsylvania State Veterinary Medical Association . . . . . . . . . . . . . . . 700–708
AMONG THE COLLEGES:
Ontario Veterinary College—Detroit Veterinary College—Harvard University, Veterinary Department—New York College of Veterinary Surgeons—Veterinary Department of the University of California . . . . . . 708–709
GLEANINGS—FROM MANY SOURCES—PERSONALS—NEW INVENTIONS . . 710–716

